# Efficient Dye-Sensitized Solar Cells Made from High Catalytic Ability of Polypyrrole@Platinum Counter Electrode

**DOI:** 10.1186/s11671-015-1015-z

**Published:** 2015-08-14

**Authors:** Xingping Ma, Gentian Yue, Jihuai Wu, Zhang Lan

**Affiliations:** Key Laboratory of Photovoltaic Materials of Henan and School of Physics & Electronics, Henan University, Kaifeng, 475004 China; Institute of Material Physical Chemistry, Huaqiao University, Quanzhou, 362021 China

**Keywords:** Polypyrrole, Platinum, Dye-sensitized solar cell, Photo-electric conversion efficiency

## Abstract

Polypyrrole@platinum (PPy@Pt) composite film was successfully synthesized by using a one-step electrochemical method and served as counter electrode (CE) for efficient dye-sensitized solar cells (DSSCs). The PPy@Pt CE with one-dimensional structure exhibited excellent electrocatalytic activity and superior charge transfer resistance for I^−^/I_3_^−^ electrolyte after being the cyclic voltammetry and electrochemical impedance spectroscopy tested. The photocurrent-photovoltage curves were further used to calculate the theoretical photoelectric performance parameters of the DSSCs. The DSSC based on the PPy@Pt CE achieved a remarkable power conversion efficiency of 7.35 %, higher about 19.9 % than that of conventional Pt CE (6.13 %). This strategy provides a new opportunity for fabricating low-cost and highly efficient DSSCs.

## Background

Since the report of dye-sensitized solar cells (DSSCs) in 1991 have attracted considerable attention due to their simple fabrication process, low production costs, relatively high conversion efficiency, and being environmental friendly [1–4]. So far, the highest photo-electric conversion efficiency of DSSCs has achieved of over 13 % [5] by depositing platinum (Pt) on a transparent conductive substrate. However, since Pt is a kind of limited resource to induce increase in cost and hider commercialize application, replacement or reduction of Pt has emerged as an important issue for further development of DSSCs.

As alternative cost-efficient materials, various counter electrode (CE) materials including carbon-based materials, conducting polymers, sulfides, nitrides, and carbides have been integrated into DSSCs [6–15]. Polypyrrole (PPy) has attracted much research attention due to its high conductivity, low cost, large electrochemical surface area, and good electrocatalytic activity for I_3_^−^ reduction enabling application in electronics, catalysis, energy storage, and sensing [6–8]. Wu et al., [16] have prepared PPy nanoparticles and applied as CE catalyst in DSSCs and got remarkable power conversion efficiency. Yue et al. [17] reported a composite CE composed of poly (3, 4-ethylenedioxythiophene):polystyrenesulfonate and PPy by using electrochemical polymerization route, which showed a good catalytic ability in I^−^/I_3_^−^ electrolyte and an improved photovoltaic performance for DSSC.

At present, although several groups have developed some alternative efficient Pt-free materials for DSSCs, Pt CE still is the most excellent and stable catalytic material. Also, a one-step route for synthesizing high-quality Pt-based hybrids as enhanced platform for electroanalytical applications is rarely a concern. Thus, in this report, we have developed a low temperature method and efficiently synthesized PPy@Pt hybrid counter electrode using one-step electrochemical deposition route, by which the obtained PPy@Pt hybrid counter electrode would exhibit excellent electrocatalytic activity and high conductivity served for DSSCs. The electrochemical performance of the PPy@Pt hybrid counter electrode were investigated by cyclic voltammetry (CV), electrochemical impendence spectroscopy (EIS), and Tafel polarization and showed excellent electrocatalytic activity and lower charge transfer resistance of 2.47 Ω·cm^2^. The DSSC with PPy@Pt CE exhibited an enhanced photovoltaic conversion efficiency of 7.35 % under irradiation of 100 mW·cm^−2^ (AM 1.5 G).

## Methods

### Preparation of PPy@Pt Hybrid Electrode

Briefly, the PPy@Pt hybrid electrode was prepared by using the electrodeposition method which outlined below. All experiments were implemented in a three-electrode cell, including one Pt foil as CE, one Ag/AgCl electrode as reference electrode, and fluorine-doped tin oxide (FTO) glass with an exposed area of 1 cm^2^ as working electrode. The base electrodeposition solution consisted of 0.1 M of pyrrole, 0.1 M of lithium perchlorate, and 0.1 M of oxalic acid in 50 ml deionized water and treated by ultrasonication for 30 min. Then, the prepared of 0.01 M chloroplatinic acid isopropanol solution was mixed into the above PPy base polymerization solution by ultrasonication for 1 h. A constant current density of 10 mA·cm^−2^ was served for electrodeposition. The obtained PPy@Pt hybrid electrode was put into anhydrous ethanol for 2 h and vacuum oven at 100 °C for 12 h, respectively. For comparison, the PPy and Pt CEs were prepared using similar method as well as the PPy@Pt hybrid electrode.

### Fabrication of DSSC

The TiO_2_ anode was prepared as described previously [18, 19]. The dye was loaded by immersing the TiO_2_ anode in the 0.3 mM of Z907 ethanol solution for 12 h. Thus, the dye-sensitized TiO_2_ anode with thickness of 8–10 μm was obtained. The DSSC was fabricated by injecting the liquid electrolyte (0.05 M of I_2_, 0.1 M of LiI, 0.6 M of tetrabutylammonium iodide, and 0.5 M of 4-tertpbutylpyridine in acetonitrile) into the aperture between the dye-sensitized TiO_2_ electrode and the CE. The two electrodes were clipped together and wrapped with thermoplastic hot-melt Surlyn.

### Characterization

The surface morphology of the sample was observed by using JSM-7600F field emission scanning electron microscope (SEM). CV, EIS, and Tafel polarization curves were conducted by using a computer-controlled electrochemical analyzer (CHI 660D, CH Instrument). The electrolyte used in the DSSC test was also injected into the symmetric dummy cells for both EIS and Tafel measurements. EIS was carried out under the simulating open-circuit conditions at ambient atmosphere, sealing with thermoplastic hot-melt Surlyn and leaving an exposed area of 0.64 cm^2^. The frequency of applied sinusoidal AC voltage signal was varied from 0.1 to 10^5^ Hz, and the corresponding amplitude was kept at 5 mV in all cases. The photovoltaic test of DSSC with an exposed area of 0.4 × 0.7 cm^2^ was carried out by measuring photocurrent-photovoltage (*J-V*) character curve under white light irradiation of 100 mW·cm^−2^ (AM 1.5 G) from the solar simulator (XQ-500W, Shanghai Photoelectricity Device Company, China) in ambient atmosphere.

## Results and Discussions

### Preparation of Nanocomposite Electrode and its Surface Morphology

**Fig. 1:**
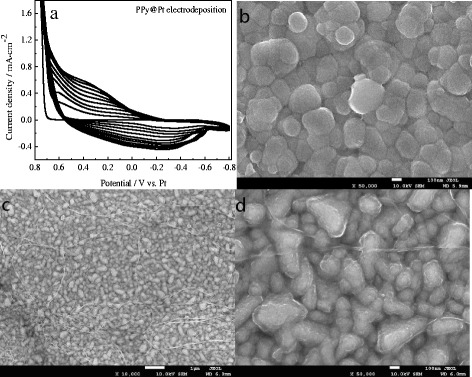
Cyclic voltammograms for the PPy@Pt coated on FTO substrate with scan rate of 30 mV·s^−1^ **(a)**; the SEM images of PPy **(b)** and PPy@Pt hybrid electrode **(c**, **d)**

Figure 1a shows the CV curves of PPy@Pt deposition on FTO substrate with scan rate of 30 mV·s^−1^. It can be seen that a pair broad anodic and cathodic peaks exhibit corresponding to the oxidation-reduction reaction of PPy@Pt CE; and the peak current densities gradually and regularly grew with the number of scans increasing for the formation of PPy@Pt conductive and electroactive materials on the substrate [20]. Figure 1b shows the SEM image of PPy film, which shows spherical-like structure and disorderly coat on the FTO. Compared with PPy film, the PPy@Pt interestingly changed conical and shuttle-like nanoparticles and uniformly deposited on the substrate with few nanowires surrounded as shown as in Fig. 1c, d. Such morphology for CE would be favorable to obtain efficient electron transfer and high electrocatalytic activity, which is crucial for the photovoltaic performance of the DSSCs.

### Electrochemical Properties

**Fig. 2:**
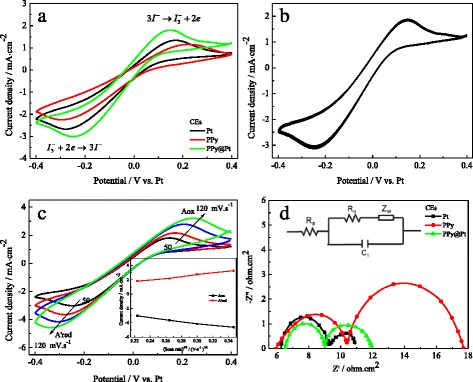
Cyclic voltammograms and EIS. CVs of the Pt, PPy, and PPy@Pt CEs at scan rate of 50 mV·s^−1^ **(a)**; the stability of the PPy@Pt CE for 40-cycle CVs **(b)**; CVs for the PPy@Pt electrode with different scan rates and the redox peaks current versus square root of scan rates **(c)**; EIS of the Pt, PPy, and PPy@Pt CEs for I^−^/I_3_^−^ redox couple **(d)**

Figure 2a presents the CVs of the various CEs at scan rate of 50 mV·s^−1^ in I^−^/I_3_^−^ electrolyte. The redox peak in the left is related to the reaction of I_3_^*−*^ + 2e^*−*^→3I^*−*^, which occurs at the CE of the DSSC and is vital for its operation; the right peaks corresponding to 3I^*−*^→I_3_^*−*^ + 2e^*−*^ and is insignificant in the context of DSSC [21]. Thus, the cathodic peaks current density (*I*_pc_) and potential (*V*_pc_) are directly proportional to the ability of the CE to reduce the I_3_^*−*^ species. The absolute value of *I*_pc_ for the Pt, PPy, and PPy@Pt CEs follows the orders of PPy@Pt (3.01 mA·cm^−2^)>Pt (2.66 mA·cm^−2^)>PPy (2.23 mA·cm^−2^); and |*V*_pc_| with the order of PPy@Pt CE (0.24 V)<Pt CE (0.27 V)<PPy CE (0.30 V). This indicates that the PPy@Pt CE owns an improved conductivity and electrocatalytic ability compared to that of the pure Pt CE or PPy CE due to their synergistic effect of the Pt and PPy. Figure 2b emerges little changed for anodic and cathodic current densities of PPy@Pt CE during the 40 times continuous cycle scans at scan rate of 50 mV·s^−1^, revealing that the PPy@Pt CE is with excellent electrochemical stability and reversibility [22]. Figure 2c shows the CVs of the PPy@Pt CE with different scan rates. It is distinctly that the cathodic peaks gradually and regularly shifted negatively, and the corresponding anodic peaks shifted positively with increasing scan rates. The current density versus (scan rate)^1/2^ plots has a good linear relationship as illustrated in the inset of Fig. 2c. This phenomenon indicates that the adsorption of iodide species is almost no effect by the redox reaction on the PPy@Pt CE surface, indicating no specific interaction between the I^*−*^/I_3_^*−*^ redox couple and the PPy@Pt CE as like Pt electrode [23, 24]. Simultaneously, the diffusion coefficient (*D*_*n*_) of CE can be estimated and listed in Table 1 following the Randles-Sevcik equation:

$$ {I}_{\mathrm{p}c}=K{n}^{1.5}\mathrm{AC}{\left({D}_n\right)}^{0.5}{v}^{0.5} $$

where *K* is the constant of 2.69 × 10^5^; *n* means the number of electrodes contributing the charge transfer (here *n* = 2); *A* is the area of the CE; *C* and *v* represent the concentration of I_3_^−^ species and the scan rate, respectively.

**Table 1:** EIS parameters of the dummy cells and the photoelectric properties of the DSSCs obtained from the Pt, PPy, and PPy@Pt CEs

Electrodes	*R*_s_ (Ω·cm^2^)	*R*_ct_ (Ω·cm^2^)	*Z*_*w*_ (Ω·cm^2^)	*V*_oc_ (V)	*J*_sc_ (mA·cm^−2^)	FF	PCEs (%)
Pt	6.21	3.11	1.65	0.70	14.12	0.62	6.13
PPy	5.71	4.69	7.35	0.72	12.77	0.55	5.06
PPy@Pt	6.53	2.47	2.94	0.73	15.50	0.65	7.35

*V*_*oc*_ open circuit voltage, *FF* fill factor, *J*_*sc*_ short-circuit current density

Thus, from Table 1, the diffusion coefficient of I_3_^−^ for the PPy@Pt CE is much larger than that of the Pt and PPy CEs, presumably deriving from its improvement of the surface roughness and the synergistic catalytic effect of Pt and PPy. Figure 2d exhibits the Nyquist plots of the symmetrical Pt, PPy, and PPy@Pt CEs to further investigate the electrocatalytic ability for regeneration of the I^*−*^/I_3_^*−*^ redox couple on the aforementioned CEs. The equivalent circuit model illustrates as inset, in which the high-frequency intercept on the real axis represents the series resistance (*R*_s_); the semicircle at high frequency refers to the charge-transfer resistance (*R*_ct_) for the I_3_^−^ reduction at the CE|electrolyte interface, and the semicircle at low frequency represents the Nernst diffusion impedance (*Z*_*w*_) corresponding to the diffusion resistance of the I^−^/I_3_^−^ redox species [25]. As everyone knows, the CE with the smaller *R*_ct_ means the less overpotential for an electron transferring from the CE to the electrolyte and great electrochemical ability. The *R*_ct_ for the abovementioned CEs are 3.11, 4.69, and 2.47 Ω·cm^2^, respectively. Among them, the PPy@Pt CE exhibits the lowest *R*_ct_ value of 2.47 Ω·cm^2^ compared to the pristine Pt electrode or PPy electrode, revealing a synergistic effect of Pt and PPy on the improvement of electrocatalytic activity and electrical conductivity for the hybrid CE. Additionally, it should be noted that the *Z*_*w*_ for the PPy CE (7.35 Ω·cm^2^) is larger than that of the Pt electrode (1.65 Ω·cm^2^). This can be attributed to the conductive polymers relatively low electrical conductivity compared with that of the Pt catalyst.

### Photovoltaic Performance of DSSCs With PPy@Pt CE

**Fig. 3:**
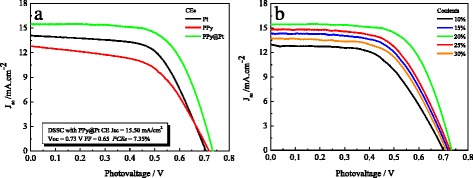
*J-V* characteristics of the DSSCs fabricated with the Pt, PPy, and PPy@Pt CEs under the standard illumination **(a, b)**

Figure 3 shows the *J-V* curves for DSSCs based on various CEs under the irradiation of a simulated solar light with an intensity of AM 1.5 G, and the derived photovoltaic parameters are summarized in Table 1. The DSSCs with PPy and Pt CEs generate *J*_sc_ of 12.77 and 14.12 mA·cm^−2^ and *V*_oc_ of 0.72 and 0.70 V, corresponding to the power conversion efficiencies (PCEs) of 5.06 and 6.13 %, respectively. Comparatively, the DSSC with PPy@Pt CE generates much better photovoltaic performances, with *J*_sc_, *V*_oc_, fill factor (FF), and PCE of 15.50 mA·cm^−2^, 0.73 V, and 7.35 %, respectively. Simultaneously, it is also discussed that the PCEs of DSSC based on the PPy@Pt CE with various contents of chloroplatinic acid. The highest PCE of the DSSC is obtained based on the PPy@Pt CE (7.35 %) with chloroplatinic acid content of 20 wt%. When the chloroplatinic acid content further increases to 25 or 30 wt%, it holds a negative influence on photovoltaic performances. The performance improvement mainly benefits from the obviously enlarged *J*_sc_, as well as the slightly increased *V*_oc_ and FF. A comparatively enhanced efficiency of the synergistic effect in the device based on the PPy@Pt hybrid CE will definitely increase the electrocatalytic ability and charge transfer, which has already revealed in the CV and EIS analyses.

## Conclusions

In this paper, we reported an efficient PPy@Pt composite film as the CE in DSSCs with power conversion efficiency of 7.35 %, which is superior to that of Pt electrode under the same conditions. The PPy@Pt CE demonstrated amazing electrocatalytic activity for the I^−^/I_3_^−^ redox reaction due to its high cathodic current density in the extensive electrochemical analyses made from CV measurement and the low *R*_ct_ of 2.47 Ω·cm^2^ from EIS test. The CE with shuttle-like structure nanoparticles prepared by using electropolymerization technique is an effective strategy for accelerating the charge transfer and iodide redox. The research presented here is far from being optimized but these profound advantages along with low-cost synthesis and scalable materials promise the new PPy@Pt CE to be a great potential and strong candidate in robust DSSCs.
